# Hypertensive Disorders of Pregnancy and Subsequent Cardiovascular Disease: Current National and International Guidelines and the Need for Future Research

**DOI:** 10.3389/fcvm.2019.00055

**Published:** 2019-05-17

**Authors:** David T. Gamble, Bolanle Brikinns, Phyo Kyaw Myint, Sohinee Bhattacharya

**Affiliations:** ^1^Ageing Clinical and Experimental Research Team, Institute of Applied Health Sciences, University of Aberdeen, Aberdeen, United Kingdom; ^2^Obstetric Epidemiology Group, Institute of Applied Health Sciences, University of Aberdeen, Aberdeen, United Kingdom

**Keywords:** pre-eclampsia, hypertensive disorder of pregnancy, guidelines, cardiovascular disease, cardiovascular disease in women

## Abstract

**Background:** It is well-established that hypertensive disorders of pregnancy (HDP) are associated with an increased risk of cardiovascular disease (CVD) in later life. National and International guidelines recognize this but due to a lack of research in this area few details are provided on how best to risk stratify or when and how to monitor these women.

**Objectives:** This article aims to summarize current guidelines in this area in order to raise awareness of need for further research in this important clinical area.

**Search Strategy:** A review of the published literature was carried out in August 2018 using the databases EMBASE and Medline and the websites of professional societies were searched manually using the search terms “pre-eclampsia,” “hypertensive disorders of pregnancy,” “management,” “guidelines,” “long term follow up” and “cardiovascular risk.” Guidelines published in English were included and articles that provided guidance on follow up post-partum of women with HDP.

**Main Results:** The search identified 360 records. Of these, 16 guidelines mentioned the follow up of women with HDP; their reported years ranges from 2010 to 2018. Only 8 (50%) provided some level of recommendation for follow up beyond the immediate post-partum period. These recognized the future risk of CVD to women from HDP and provide detailed recommendations for the management of these conditions during pregnancy and in the immediate post-partum period. Guidelines recommended that women and primary care clinicians are made aware of this risk and some suggest yearly BP monitoring, and at least 5 yearly monitoring of renal functions, urinalysis and lipid profile testing alongside lifestyle modifications and control of CVD risk factors. Guidelines used a combination of meta-analysis, individual cohort studies and expert opinions to inform their recommendations.

**Conclusions:** There is a need for future studies of women with a history of HDP to define their trajectory for the development of CVD and candidate biomarkers in order to develop screening, risk stratification, and preventive measures to reduce the significant CV burden associated with HDP in women.

## Introduction

Historically, hypertensive disorders of pregnancy (HDP) were believed to be self-limiting with little effect on health once blood pressure (BP) had returned to normal in the postnatal period. It is now well-established that this group of conditions, comprising gestational hypertension, pre-eclampsia and eclampsia, are associated with an increased risk of cardiovascular disease (CVD) in later life (1–3). CVD is one of the most important causes of death in women and has a huge impact on healthcare costs; in the UK the NHS spent more than £5.9 billion on heart disease between 2013 and 2014 (4).

Pre-eclampsia in particular has been shown in a number of systematic reviews and meta-analyses to impact on the health and well-being of women extending beyond pregnancy outcomes. A systematic review and meta-analysis in women with prior pre-eclampsia showed the relative risks [(95% CI) for heart failure (HF) and CVD death to be 4.19 (2.09–8.38) and 2.21 (1.83–2.66), respectively (1)]. In another systematic review and meta-analysis the relative risks (95% CI) in women with prior pre-eclampsia were 3.70 (2.70–5.05) for hypertension (HT), 2.16 (1.86–2.52) for ischaemic heart disease (IHD) and 1.81 (1.45–2.27) for stroke (2).

Some national and international guidelines recognize these future risks and recommend routine follow up of women who had suffered from HDP in order to prevent heart disease. However, due to a lack of research in this area, few details are provided on how best to do this. This article aims to summarize current guidelines in this area in order to raise awareness of this important clinical uncertainty.

### Search Strategy/Methodology

**Figure 1:**
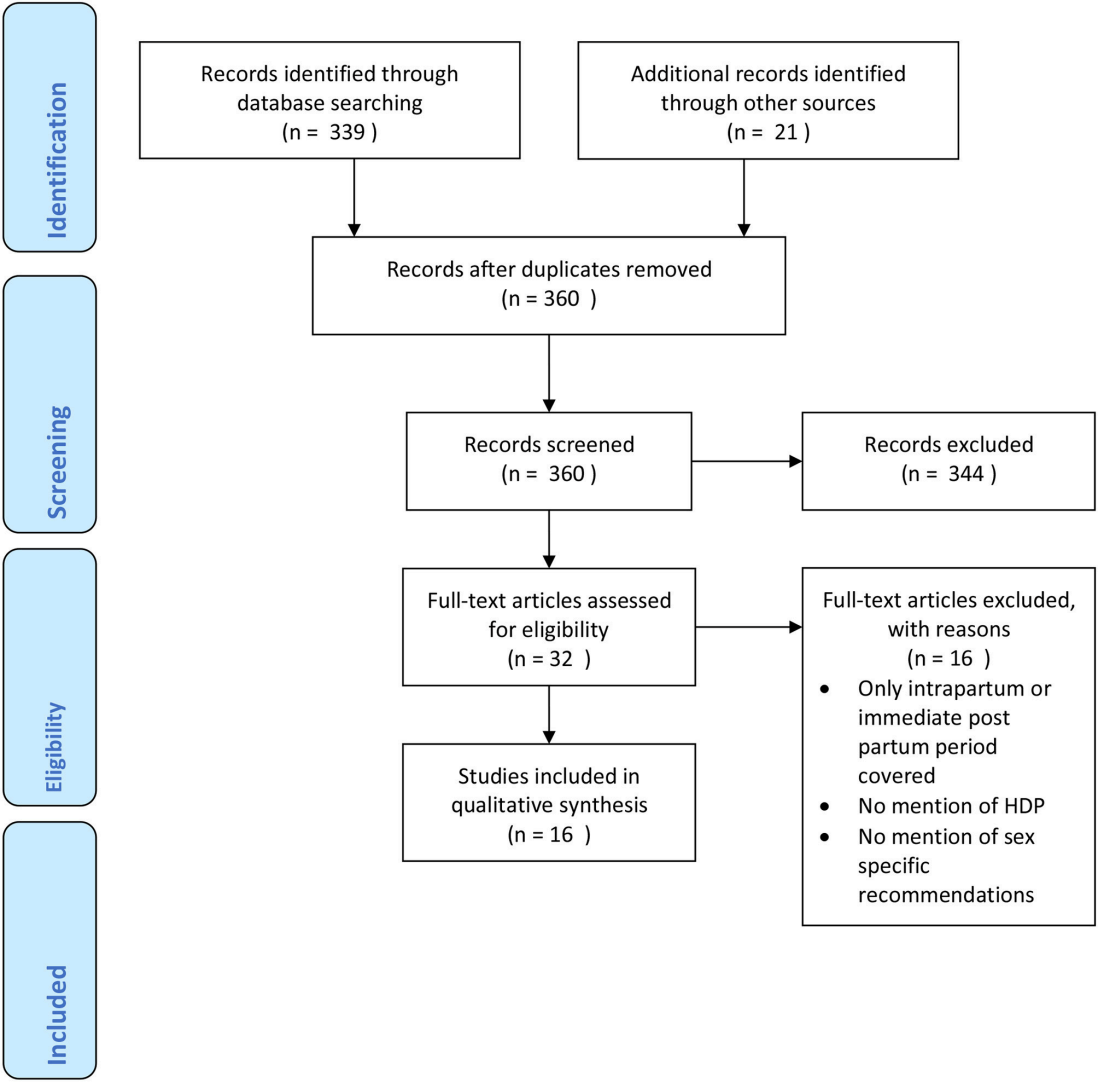
Prisma flow chart of guideline inclusion.

A review of the published literature was carried out using the databases EMBASE (1980–2018) and Medline (1946–2018) and the websites of relevant professional societies such as The National Institute for health and Care Excellence (NICE), The European Society of Cardiology (ESC), The Institute of Obstetricians and Gynecologists of Ireland and The American college of Obstetricians and Gynecologists were hand-searched in August 2018. The search was undertaken using the search terms “pre-eclampsia,” “hypertensive disorders of pregnancy,” “management,” “guidelines,” “long term follow up” and “cardiovascular risk.” All of the relevant articles returned were published between 2010 and 2018. Reference lists of identified articles were also scrutinized. Guidelines published in English (or with English translation) were included. Articles that provided guidance on follow up of women with HDP post-partum period were selected. The relevant data were collected from the published full text. Databases were last searched on 5th August 2018. The Prisma flow chart for guideline inclusion is shown in Figure 1.

Summary tables of identified guidelines were developed to outline details on referral to specialist teams, recommendations for investigations, monitoring frequency and follow up time and any other recommendations for risk factor modification or preventative actions. The guidance was also scrutinized for the level of evidence upon which these recommendations were based and assigned a final grade for the quality of evidence as “high,” “moderate,” “low,” or “very low” based on the strength and quality of underlying evidence for the critically important outcomes based on the Grading of Recommendations Assessment, Development, and Evaluation (GRADE) principles. This is also summarized.

## Results

Guidelines were summarized in Table 1, their recommendations were divided specialist referral, recommendations for investigations and monitoring frequency and follow up time and risk factor modification and other preventative actions. The graded level of underlying evidence was also specified. The search identified 360 records. Of these, 16 guidelines mentioned the follow up of women with HDP; their reported year ranges from 2010 to 2018. Only 8 (50%) provided some level of recommendation for follow up beyond the immediate post-partum period.

**Table 1:** Summary of international guidelines with the level of evidence for individual recommendations.

**Guideline**	**Year**	**Specialist referral (graded level of evidence)**	**Recommendations for Investigations and monitoring frequency and follow up time (graded level of evidence)**	**Risk factor modification and other preventative actions (graded level of evidence)**	**Quality of evidence (based on GRADE principles)**
National Institute for health and Care Excellence (NICE) clinical guideline 107 (5).	2011	Those who have no proteinuria and are normotensive at the postnatal review require no further renal follow-up (2^b^).	Thrombophilia screening is not indicated (2^a^).	Women should be told, along with their primary care physicians, that these conditions are associated with an increased risk of developing CVD (3a−)^*1^. Women should maintain a BMI between 18.5 to 24.9 kg/m2, in line with NICE clinical guideline 43 (2^b^).	Moderate.
National Collaborating Center for Women's and Children's Health (UK). Hypertension in Pregnancy: The Management of Hypertensive Disorders During Pregnancy (6).	2010	Not specified.	Further follow-up is necessary but unsatisfactory evidence to support recommendations on frequency of follow up or BP monitoring (3^a^).	Inform women and their primary care clinicians of the possibilities of developing high BP and its complication in the future (3a).	Low.
ESC/ESH Guidelines for the management of arterial hypertension The Task Force for the management of arterial hypertension of the European Society of Cardiology (ESC) and the European Society of Hypertension (ESH) (7).	2018	Not specified.	Recommends annual visits to a primary care physician for BP checks and to check other metabolic factors (2^a^). Recommends long-term specialist follow-up.	Previous hypertension in pregnancy or pre-eclampsia should be part of a clinical history. Lifestyle modifications are indicated to reduce future cardiovascular risk (2^a^).	low.
The European Society of Cardiology guideline on the management of cardiovascular diseases during pregnancy (8).	2011	Not specified.	Regular BP monitoring and control of metabolic risk factors (3a-)^*4^.	Lifestyle changes to minimize difficulties in future pregnancies and reduce the possibilities of developing cardiovascular disease in the future (2a−)^*5^.	Low.
Institute of Obstetricians and Gynecologists Ireland Clinical Practice guideline No. 3 (9).	2016	All patients with severe pre-eclampsia to be offered hospital follow-up within 12 weeks of delivery (5).	Blood pressure and proteinuria assessment should be carried out and specialist referral made if there is ongoing hypertension, need for antihypertensives or significant proteinuria (5).	Discuss potential risk factors such as obesity and aspirin therapy (5).	Very low.
Institute of Obstetricians and Gynecologists Ireland Clinical Practice guideline No. 37. (10).	2016	Further care after 6 weeks for any ongoing pregnancy related changes, in particular chronic high blood Pressure, ongoing need for antihypertensives, high BMI or incidence of pre-term pre-eclampsia (5). Provide expert review if still on antihypertensive medicines by 6–8 weeks (5).	Yearly BP and standard cardiovascular risk assessment including serum lipids and blood glucose (5). Women with persistent hypertension should undergo treatment and investigation in line with standard protocols (5).	Psychotherapy for women with history of had hypertensive disorders in pregnancy to promote their well-being and lifestyle advice including avoiding smoking, maintaining a healthy body mass, engaging in regular exercise and maintaining a balanced diet (5).	Very low.
Hypertension and Pregnancy: expert consensus statement from the French Society of Hypertension, an affiliate of the French Society of Cardiology (11).	2017	Women should be reviewed by a consultant to ensure that CVD and renal disease risk factors are identified and controlled (2^a^). Assessment and management of all CVD and renal risk factors should be offered to all women via a multidisciplinary care-plan (2^a^).	Women with a known past medical history of high BP during pregnancy should undergo BP (3^a^), renal function and urinalysis monitoring (2^a^).	Highlights the importance of a multi-disciplinary approach in monitoring and ensuring a healthy life style and modulation of CVD risk factors (5). Women should be provided with information concerning the possibilities of developing high BP and its complication in the future (3^a^).	Low.
Promoting Risk Identification and Reduction of Cardiovascular Disease in Women Through Collaboration With Obstetricians and Gynecologists: A Presidential Advisory From the American Heart Association and the American College of Obstetricians and Gynecologists (12).	2018	Recommendations are given for all women and not specific to those with HDP.	Blood pressure should be checked yearly for those ≥40 years or those with increased risk for high blood pressure (including HDP) (2^a^).	Recommendations are given for all women and not specific to those with HDP.	Low.
ACC/AHA/AAPA/ABC/ACPM/AGS/APhA/ ASH/ASPC/NMA/PCNA Guideline for the Prevention, Detection, Evaluation, and Management of High Blood Pressure in Adults A Report of the American College of Cardiology/American Heart Association Task Force on Clinical Practice Guidelines (13).	2017	Not specified.	No evidence that blood pressure thresholds, blood pressure targets, treatment choices or antihypertensive combinations should differ in women compared to men (1^a^).	Not specified.	Moderate.
The American College of Obstetricians and Gynecologists Task Force on Hypertension in Pregnancy (14).	2013	Not specified.	Yearly BP and risk factor monitoring (such as lipids, fasting blood glucose and BMI) suggested for women with medical history of recurrent pre-eclampsia and pre-term pregnancy (< 37 weeks) (5).	Women should maintain a healthy lifestyle in terms of maintaining an optimum body weight, consuming a diet high in fiber, fruit and vegetables and low in fat and avoid tobacco (5). Evaluate the future risk of cardiovascular disease (2^a-^)^*6^.	Very low.
Effectiveness Based Guidelines for The Prevention of CVD in Women: A Guideline From the American Heart Association (15).	2011	Women should be referred post-partum to a primary care physician or cardiologist to aid future care plan and manage risk factors (3^a-^)^*1^.	Not specified	Emphasizes the need for female-based guidelines (5). Hypertensive disorders of pregnancy should be part of a detailed cardiovascular history (3^a-^)^*1^.	Very low.
The Association of Ontario Midwives clinical practice guideline 15 (16).	2012	Thorough examination post-partum period within 4 weeks and further post-partum visits or clinician consultation if clinical manifestations of HDP beyond 4 weeks (3^b^). Communication between clinicians and community healthcare providers on future blood pressure care (3^a^).	Not specified	Inform women of their risk of developing pre-eclampsia in future pregnancies and Information about hypertensive disorders of pregnancy should be passed onto primary care physicians (3^a-^)^*3^. Dietary and lifestyle changes—exercise activity, reducing fat and salt intake to reduce high BP at the later life recommended for pregnant women (5).	Very low.
The Guidelines for the Prevention of Stroke in Women, a Statement for Healthcare Professionals from the American Heart Association and American Stroke Association (17).	2014	Review all women from 6 TO 12 months post-partum and menopausal women and record history of preeclampsia/eclampsia as a risk factor (3a−)^*7^.	Lipids levels should be tested (1c−^*8^).	Evaluate and treat for cardiovascular risk factors such as high BP (3a−^*7^), overweight women, smoking and elevated lipids levels (1c-)^*8^.	Moderate.
Society of Obstetricians and Gynecologists of Canada Clinical Practice Guideline 307 (18)	2014	Referral to internal or renal medicine should be considered in women with refractory post-partum hypertension or indicators of renal disease beyond 3–6 months (3^a^).	Women with underlying hypertension or persistent postpartum hypertension should undergo urinalysis, renal function and electrolytes, fasting glucose, fasting lipids and 12-lead electrocardiography at least 6 weeks post-partum (3a). Women who are normotensive at discharge may benefit from assessment of cardiovascular risk factors and women with a history of severe pre-eclampsia should be screened for underlying hypertension or renal disease (2^a^)	All women who have had a hypertensive disorder of pregnancy should maintain a healthy diet, lifestyle and BMI (2^a^)	Low.
The SOMANZ Guideline for the Management of Hypertensive Disorders of Pregnancy (19)	2014	Not specified	Women should have an annual BP check and 5 yearly assessment of CVD risk factors including serum glucose and lipid profiles (5)	Women would benefit from a healthy lifestyle that included a healthy weight, not smoking, exercise and a healthy diet (3a−)^*2^.	Very low.
The Queensland Maternity and Neonatal Clinical Guidelines Program Guideline No. MN10.13.V4-R15 (20)	2010	Not specified	Women should be screened for pre-existing hypertension and underlying renal disease (2a) Cardiovascular risk factors (e.g., BP, lipid profile and serum glucose) should be assessed regularly with patient-centered follow up time (3^a^).	Post-natal counseling should include consultation on risk factors and preventative therapies such as calcium supplementation and low dose aspirin (3^a^). Women should maintain a healthy lifestyle in terms of diet, exercise and avoidance of tobacco (3^a^−^*9^).	Low.

Minus plus

* *indicated were there are issues with scoring the level of evidence as listed below such as heterogeneity/or has stated below*.

*1 *Heterogeneity I^2^ = 62.6% for increased risk of future hypertension with evidence of small study projecting larger effects size. Low heterogeneity for Stroke = I^2^ = 0% for Stroke (no evidence of small study bias P = 0.82) and IHD and I^2^ = 27.1% (no evidence of small study bias P = 0.59). More recent paper in 2008 uses Systematic review on cohort with two additional cohorts and case control studies and scores heterogeneity I^2^ scores ranging from 35.7 to 66.3%. Evidence also measures the severity of pre-eclampsia and CVD Risk using meta regression*.

*2 *Similar papers on meta-analysis/systematic review used on lifestyle factors as annotated 1*.

*3 *Same papers used as evidenced for annotated 1*.

*4 *One of the evidence used focuses on maternal placental syndrome/poor fetal growth and no measurement on weight and HBP to reduce bias on obese women*.

*5 *Systematic reviews examined for both randomized and large prospective cohort studies*.

*6 *Clear evidence with pre-eclampsia and future CV, however the significance and applicable stages are not established*.

*7 *Case control Dutch study and Cohort/systematic reviews similar papers as annotated 1*.

*8 *Lipids in obese women doesn't have a clear focus on pregnant women*.

*9 *Referenced Somanz guideline as per annotated 2, same evidence applies as annotated 1*.

**Level of evidence 1:**

*1a,Systematic reviews (with homogeneity) of randomized controlled trials;1b,Individual randomized controlled trials (with narrow confidence interval); 1c, All or none randomized controlled trials;2a, Systematic reviews (with homogeneity) of cohort studies; 2b, Individual cohort study or low quality randomized controlled trials (e.g., < 80% follow-up);2c, “Outcomes” Research; ecological studies; 3a, Systematic review (with homogeneity) of case-control studies; 3b, Individual case-control study; 4, Case series (and poor-quality cohort and case-control studies); 5, Expert opinion without explicit critical appraisal, or based on physiology, bench research or “first principles;” (21)*.

### Summary of Guidelines

#### UK

The National Institute for health and Care Excellence (NICE) have issued clinical guideline 107 in 2011, titled Hypertension in pregnancy: diagnosis and management (5). This guideline provides detailed recommendations for the diagnosis and management of HDP during the pregnancy, intrapartum and immediate post-partum period. The guidance for follow up and ongoing intervention following discharge is focused specifically on those women who remain hypertensive or on hypertensive medication. For these women, it recommends that information should be provided to primary care including who will provide medical review, frequency of BP monitoring and thresholds for reducing or stopping treatment, although no specific details are given. It also provides guidance on frequency of post-natal and medical reviews for women who remain hypertensive and suggests hematological and biochemical monitoring for those with deranged blood tests on discharge or those who remain proteinuric. It states that women who have had pre-eclampsia should be offered a medical review at the 6–8 week postnatal review, those who are still on antihypertensive treatment 2 weeks after discharge should be offered a medical review and those who still need antihypertensive treatment at the postnatal review should be offered a specialist assessment of their hypertension. However, whilst this guideline states that women who have had gestational hypertension or pre-eclampsia should be informed along with their primary care physicians that these conditions are associated with an increased risk of developing hypertension and its complications in later life, no details are provided on how to monitor these women once they are off their antihypertensive medication and their BP has normalized. It does nonetheless recommend that women with a history of pre-eclampsia who have no proteinuria and are normotensive at the postnatal review require no further renal follow-up or thrombophilia screening and that they should maintain a BMI between 18.5 and 24.9 kg/m^2^, in line with NICE clinical guideline 43 titled Obesity prevention (22). NICE used a combination of individual observational cohort and case control studies to inform their recommendations. NICE are currently updating their recommendations in this guideline including their advice on follow up care after transfer to community care. This updated guidance is expected to be published in June 2019. The specific recommendation in HDP have not been made available at the time of writing.

In 2010, The National Collaborating Centre for Women's and Children's Health (UK) issued a guidance titled “Hypertension in Pregnancy: The Management of Hypertensive Disorders During Pregnancy” (6). This echoes NICE CG 107 by recommending women and their primary care doctors to be informed of the future CVD risk. It highlights that there is insufficient evidence for practitioners to provide recommendations on the frequency of follow up. These Guidelines used meta-analysis from Bellamy et al. (2) and Macdonald et al. (3) for their recommendations as well as cohort studies by Wilson et al. (23).

#### Europe

In 2018 the ESC issued guidelines for the management of arterial hypertension (7). This recommends annual visits to a primary care physician for BP checks and to other metabolic risk factors. It also suggests previous hypertension in pregnancy or pre-eclampsia should be part of a clinical history and lifestyle modifications are indicated to reduce the risk of CVD in the future.

The ESC has issued guidance on the management of CVDs during pregnancy (8). Beyond the immediate post-partum period, it recommends lifestyle modifications, regular BP review and control of metabolic factors to mitigate maternal cardiovascular risk in the future. These Guidelines used meta-analysis from Macdonald et al. (3), cohort studies from Wilson et al. (23) and the Effectiveness-based guidelines for the prevention of CVD in women —2011 update: a Guideline from the American Heart Association as the basis for their recommendations.

The Institute of Obstetricians and Gynaecologists of Ireland have issued clinical practice guideline no. 3 in 2016, titled Diagnosis and management of severe pre-eclampsia and eclampsia (9). Again, their recommendations post-partum focuses on the immediate management of women with ongoing hypertensive needs, recommending BP monitoring every 1–2 days for up to 2 weeks after discharge until antihypertensive treatment has been discontinued and the patient is normotensive. It does suggest that, all patients with severe pre-eclampsia should be offered an appointment in secondary care within 3 months of delivery. Interestingly, the Institute of Obstetricians and Gynaecologists of Ireland provides little specific details. Other assessments, such as BP and proteinuria should also be performed. This includes referral to specialist services if antihypertensive treatment is ongoing and required or proteinuria is confirmed. It echos the NICE guidelines with regards to information that should be provided to primary care following discharge. The same institution issued The Clinical Practice Guideline no. 37 in 2016, titled the management of hypertension in pregnancy (10); this recommends that follow-up after 6 weeks post-partum is required to ensure resolution of pregnancy-related changes and to determine the need for ongoing care. It suggests those at high risk of ongoing hypertension include those with chronic hypertension, prolonged anti-hypertensive treatment, higher maximum BPs during pregnancy, higher BMI and those with pre-eclampsia that occurred preterm. Women with persistent hypertension not previously assessed should undergo routine work-up and be given advice regarding future lifestyle and optimization of risk factors in subsequent pregnancies. This includes those who are obese, have other cardiovascular risk factors, secondary hypertension or end-organ disease. They gathered evidence from Health professionals' opinions for their specialist referral, recommendations, risk factor modification, and other preventative actions.

The French Society of Hypertension, an affiliate of the French Society of Cardiology, issued an expert consensus statement on hypertension and pregnancy in 2017 (11). This recommends that, women with pre-eclampsia should be reviewed by a consultant to ensure CVD and renal disease risk factors are identified and controlled. It highlights that, regular monitoring, healthy life style, and modulation of CVD risk factors is essential to reducing CVD in the future. It also recommends communicating effectively to the patient. It suggests that, women who have had a hypertensive disorder of pregnancy should have the etiology of the disease assessed and undergo BP, renal function and urinalysis monitoring. They should also undergo long term BP monitoring, even after their BP has normalized post-delivery. They derived evidence from meta-analysis of observational studies by Bellamy et al. (2) as well as from Health professionals' opinions for their recommendations.

#### North America

In 2017 the American College of Cardiology/American Heart Association task force on clinical practice guidelines issued guidance on the prevention, detection, evaluation, and management of high blood pressure disorders in adults (13). This stated that there was no evidence that blood pressure thresholds, blood pressure targets, treatment choices, or antihypertensive combinations should differ in women compared to men but mentioned little else specific to women or HDP.

The American Heart Association and the American College of Obstetricians and Gynecologists have issued guidance titled “Promoting Risk Identification and Reduction of Cardiovascular Disease in Women Through Collaboration With Obstetricians and Gynecologists: A Presidential Advisory From” in 2018 (12). This recognizes the need for sex specific guidelines targeted to women. It provides recommendations for cardiovascular prevention including management for monitoring of hypertension, hyperlipidaemia, diabetes, and healthy lifestyle advice. However, despite listing HDP as an important risk factor for future CVD, specific recommendations for those who have suffered HDP are not provided other than yearly blood pressure checks with an increased risk for high blood pressure (including those who have suffered HDP). These recommendations are based on US Preventive Services Task Force final recommendation statement for high blood pressure in adults, which itself is based on evidence synthesis of systematic reviews of observational studies.

The American Heart Association issued a guideline in 2011 titled effectiveness-based guidelines for the prevention of CVD in women (15). This emphasizes the need for female based guidelines and an individualized approach to managing cardiovascular risk in women. In this guideline, a history of pre-eclampsia or pregnancy-induced hypertension puts women in a high risk category for CVD and it goes on to say that pregnancy provides an opportunity to estimate a woman's future cardiovascular risk, for which pre-eclampsia may be an early indicator. The guideline also states that women should be referred post-partum to a primary care physician or cardiologist to monitor and control cardiovascular risk factors and that HDP should be part of a detailed cardiovascular history in any setting. Whilst the guideline recognizes the building evidence base that pre-eclampsia is an important cardiovascular risk factor and acknowledges the need for future research in this field, it does not provide any details on how to monitor or determine the effectiveness of diagnostic and preventive interventions in this critical group. They used meta-analysis of observational studies such as Bellamy et al. (2) as the underlying evidence for their recommendations.

The American college of Obstetricians and Gynaecologists Task Force on Hypertension in Pregnancy issued a guidance titled hypertension in pregnancy in 2013. This states that pre-eclampsia, particularly if associated with pre-term delivery, is a strong risk factor for CVD. It recommends that, women should maintain a healthy lifestyle in terms of maintaining an optimum body weight, consuming a diet high in fiber, fruit and vegetables, and low in fat and to avoid tobacco. Furthermore, it states that future CVD risk factors should be considered and provides no further details regarding the timing and frequency of these evaluations. They gathered evidence from Health professionals' opinions for their recommendations on specialist referral, risk factor modification, and other preventative actions.

The Guidelines for the Prevention of Stroke in Women, a Statement for Healthcare Professionals from the American Heart Association and American Stroke Association in 2014 (17) recognizes the link between pre-eclampsia or eclampsia and CVD and stroke outcomes. It states that, “insufficient evidence exists to inform any recommendation for screening, prevention, or treatment in women with a history of pregnancy complications or adverse pregnancy outcomes.” It does however suggest that those women with ongoing hypertension should be managed according to adult guidelines. It also recommends women with a history of pre-eclampsia or eclampsia should have this documented as a risk factor and that these women should have common CVD risk factors (including smoking and dyslipidaemia) identified and treated. These guidelines used evidence from meta-analysis of cohort studies including Macdonald et al. (3) for their recommendations as well as the Wilson et al. (23) cohort study.

The Association of Ontario Midwives issued clinical practice guideline 15 in 2012, titled HDP (16). This recommends that, midwives discuss the need for healthy lifestyle choices with women post-delivery and provide information on HDP to primary care physicians. They gathered evidence from systematic reviews of observational studies including Bellamy et al. (2) and Macdonald et al. (3) as well as Health professionals' opinions for the specialist referral, recommendations, risk factor modification, and other preventative actions.

The Society of Obstetricians and Gynaecologists of Canada issued clinical practice guideline 307 titled Diagnosis, Evaluation, and Management of HDP in 2014 (18). This echos NICE clinical guideline 107 by providing details on review frequency, treatment options and BP targets for the immediate 6 weeks period post-partum. It provides some guidance on ongoing care beyond 6 weeks, which again focuses predominantly on those still requiring hypertensive medication or those with persistently raised BP or deranged renal function. It states that women with a history of severe pre-eclampsia should be screened for underlying hypertension or renal disease and that referral to internal or renal medicine should be considered in those women with refractory post-partum hypertension or indicators of renal disease (e.g., proteinuria) beyond 3–6 months. It reveals that, those women with underlying hypertension or persistent postpartum hypertension should undergo urinalysis, renal function and electrolytes, fasting glucose, fasting lipids and 12-lead electrocardiography at least 6 weeks post-partum. It also reveals that, women who are normotensive at discharge may benefit from assessment of cardiovascular risk factors and all women who have had a hypertensive disorder of pregnancy should maintain a healthy diet, lifestyle, and BMI. These guidelines used a blend of individual observational cohort and case control studies to inform their recommendations.

#### Australia and New Zealand

The SOMANZ Guideline for the Management of HDP published by the Society of Obstetric Medicine of Australia and New Zealand in 2014 (19). This recommends that, women would benefit from a healthy lifestyle which includes: a healthy weight, smoking cessation program, exercise and a healthy diet. It also recommends that, these women should have an annual BP check and 5 yearly assessments of CVD risk factors including serum glucose and lipid profiles. The SOMANZ guidelines used a literature review as their evidence for lifestyle recommendations, the literature review referenced Bellamy et al. (2) meta-analysis as their evidence. They also used expert opinions to develop their recommendations.

The Queensland Maternity and Neonatal Clinical Guidelines Program issued Guideline No. MN10.13.V4-R15 in 2010, titled HDP (20). This recommends women to undergo screening for pre-existing hypertension and underlying renal disease, cardiovascular risk factors (e.g., BP, lipid profile, and serum glucose) and the necessity of women regularly assessed. According to them, women should also maintain a healthy lifestyle with their diet choice, exercise, and avoid tobacco. The Queensland Maternity and Neonatal Clinical Guidelines recommendations for investigations and monitoring BP used The National Collaborating Centre (NCC) 2011 revised version; The NCC referenced Bellamy et al. (2)'s work, to support their recommendations.

## Discussion

This is the first summary of current international guidelines evaluating long term follow up for women who have suffered HDP. Whilst all guidelines recognize the higher risk of CVD in those with these conditions, and in the most part recommend informing women and their general practitioner or family doctors (GPs) of this, there is no consensus regarding who to monitor intensively, for how long or how frequently and what parameters should be used for screening initially and perhaps at a future at risk period. Guidelines issued by obstetrics and gynecological societies focus on the management and identification of HDP during pregnancy and the immediate postpartum period. Few details are given regarding timing and frequency of monitoring, appropriate physical, and biomarkers for longer term monitoring or strategies for the prevention of CVD in later life. Guidelines issued by cardiological societies are more explicit with some recommending yearly BP monitoring, and at least 5 yearly renal functions, urinalysis and dyslipidaemia testing. Some also recommend lifestyle modifications to achieve a healthy weight, smoking cessation, and control of glucose and lipid profiles. The most detailed recommendations for future follow up comes from the ESC, Institute of Obstetricians and Gynecologists Ireland, The American College of Obstetricians and Gynaecologists, the American Heart Association and The SOMANZ Guidelines. They recommend up to yearly BP monitoring and yearly assessment of CVD risk factors. These guidelines used the highest available level of evidence from meta-analysis as well as specialist opinions from healthcare professionals to inform their recommendations. Interestingly, the ESC guidelines also make reference to the use of N-terminal pro-B natriuretic peptide (NT-proBNP) as part of an investigative work up for patients with hypertensive emergencies, including pre-eclampsia. NT-proBNP has been shown to be strongly related to cardiovascular events (24). Measurement of such biomarkers in women with prior HDP needs further evaluation as a potential predictor or tool to risk stratify for future cardiovascular events.

Guidelines used a variety of levels evidence to inform their recommendations. Many sited expert opinions as their underlying evidence; including The Institute of Obstetricians and Gynaecologists Ireland Clinical Guidelines 2010 and 2016 and The American College of Obstetricians and Gynaecologists Task Force on Hypertension in Pregnancy (14). The report from the American College of Cardiology (2017) used prospectively designed overviews of randomized trials. The ESC and the European Society of Hypertension (ESH) used retrospective cohort studies for their recommendations. The Society of Obstetricians and Gynaecologists Canada Clinical Practice Guideline 307 (2014) and NICE (2011) used a blend of individual observational cohort and case control studies as underlying evidence to their recommendations. A number of guidelines referenced the meta-analysis of Bellamy et al. (2), Macdonald et al. (3) and cohort studies by Wilson et al. (23). These included the French Society of Cardiology (2017), the American Heart Association (2011), The Association of Ontario Midwives Clinical Practice (2012), the SOMANZ guidelines and The Queensland Maternity and Neonatal Clinical. Whilst a number of guidelines used the highest available evidence, namely the meta-analysis of observational studies by Bellamy et al. (2) and Macdonald et al. (3) these studies focus primarily on describing the association between HDP and future CVD risk. There is still little high level evidence on how or when to initiate follow up on these women and no evidence on the effectiveness of recommended strategies in this unique patient population. This clinical area falls between obstetrics and gynecology and cardiology and general practitioners are perhaps best placed to follow up these high risk women once they are discharged into the community following the birth of their baby.

This article has a number of strengths. We have systematically reviewed all current English language guidelines on this subject, capturing the majority of major international guideline committees, and it is clear that there is an overwhelming paucity ofrecommendations for how to manage women with previous HDP going forward into later life.

This article has limitations that should be discussed. We have only included English language guidelines and it is possible that guidelines exist in other languages. Nonetheless, as we have captured the major international guideline committees on this subject it is unlikely that the guidelines that exist will change the overall conclusions of this article. As this is a review of clinical guidelines not clinical practice, it is possible that individual centers have their own protocols and clinical strategies not captured here. However, this is a novel and expanding area and it is likely most clinical centers will be influenced by these guidelines. It is possible that guidelines are influenced by the opinions and clinical experience of the guideline development group and not just the available objective evidence. However, we have captured large globally influential guideline groups and their opinions should be considered relevant and expert on this subject.

We have systematically searched for guidelines published in peer reviewed journals and by major professional societies using transparent inclusion and exclusion criteria agreed *a priori*. However, these were limited to English language publications or translations only. We have summarized the relevant guidance from the clinical guidelines and evaluated the evidence underpinning the recommendations. This process has highlighted gaps and uncertainties in clinical guidance on how to manage women who have had HDP in the longer term in order to monitor and mitigate their risk of CVD. Further research needs to address the following clinical questions:

- When should we start to monitor women with HDP after being discharged in the community following normalization of their BP?
- When and how frequently should women with HDP be monitored if their BP has returned to normal?
- What investigations should form part of their monitoring to maximize the chance of early detection of CVD risk?
- What interventions are effective in reducing the risk of subsequent CVD in women with HDP?

Once we have accumulated the evidence base from robust research, clinical guidelines should follow. Clinical guidelines are important in changing practice and behavior (25) provided they are consistent and based on clear evidence.

## Conclusion

Whilst these guidelines recognize the future risk to women from HDP and deal appropriately with management of these conditions during pregnancy and in the immediate period post-partum there is a paucity of recommendations for how to manage these women going forward. There is a need for high quality studies of women with a history of HDP to define their trajectory for the development of CVD and then to develop screening, risk stratification, and preventive measures.

## Author Contributions

PM and SB conceived the study. Articles were searched by DG and BB. DG and BB drafted the paper and all of the authors contributed in writing and reviewing the paper.

### Conflict of Interest Statement

The authors declare that the research was conducted in the absence of any commercial or financial relationships that could be construed as a potential conflict of interest.
